# Antibacterial and antibiotic-modulation activity of six Cameroonian medicinal plants against Gram-negative multi-drug resistant phenotypes

**DOI:** 10.1186/s12906-016-1105-1

**Published:** 2016-05-04

**Authors:** Doriane E. Djeussi, Jaurès A. K. Noumedem, Bonaventure T. Ngadjui, Victor Kuete

**Affiliations:** Department of Biochemistry, Faculty of science, University of Dschang, P.O. Box 67, Dschang, Cameroon; Department of Organic Chemistry, Faculty of Science, University of Yaoundé I, Yaoundé, Cameroon

**Keywords:** Antibiotics, Antibacterial, Cameroon, Gram-negative bacteria, Multi-drug resistance, Synergy

## Abstract

**Background:**

Bacterial Infections involving multi-drug resistant (MDR) phenotypes constitute a worldwide health concern. The present work was designed to assess the antibacterial properties of the methanol extracts of six medicinal plants (*Anthocleista schweinfurthii*, *Nauclea latifolia*, *Boehmeria platyphylla, Caucalis melanantha, Erigeron floribundus* and *Zehneria scobra*) and the effects of their associations with antibiotics on MDR Gram-negative bacteria over-expressing active efflux pumps.

**Methods:**

The antibacterial activities and the ability to potentiate antibiotic effects of the methanol extracts the tested plants were evaluated *in vitro* against twenty eight Gram-negative bacteria expressing MDR phenotypes, using broth microdilution method. The phytochemical screening of these extracts was also performed using standard methods.

**Results:**

All tested extracts displayed moderate to low antibacterial activity on at least 14.3 % of the 28 tested bacteria, with MIC values ranged from 128 to 1024 μg/mL. The best antibacterial spectrum was observed with *Naulcea latifolia* bark extract. Extracts from *A. schweinfurthii* fruits, *N. latifolia* stem bark, *Z. scobra* and *N. latifolia* leaves showed synergistic effects with many antibiotics against MDR bacteria.

**Conclusion:**

The overall results of the present study provide information for the possible use of the studied plants, especially *Nauclea latifolia* in the control of Gram-negative bacterial infections including MDR species as antibacterials as well as resistance modulators.

**Electronic supplementary material:**

The online version of this article (doi:10.1186/s12906-016-1105-1) contains supplementary material, which is available to authorized users.

## Background

Infectious diseases still represent one of the major health concern worldwide [1]. According to the National Institute of Health, infectious diseases are the second cause of death and the leading cause of loss of productive life years worldwide. Bacterial infections are responsible of about 70 % of cases of death related to microorganisms [1]. The use of antibiotics and hygiene rules helped to fight infectious diseases in the past. However, they are becoming increasingly difficult to control as results of the spread of resistant phenotypes. The resistance to antibiotics has increased in recent decades, mainly due of their inappropriate use [2]. Bacteria have developed several mechanisms of resistance including active efflux which plays an important role in multi-drug resistance (MDR), mainly in Gram-negative bacteria [3]. There is a need for the discovery of new active antimicrobials to combat MDR microorganisms. Amongst the new areas explored to overcome infectious diseases caused by MDR bacteria, medicinal plants seem to offer an ideal alternative since they are readily available source of bioactive agents and are well accepted by about 80 % of the world population. Many African medicinal plants and their metabolites were previously found active against MDR Gram-negative bacteria [4, 5]. Also the synergistic activities of some African medicinal plants with antibiotics against MDR Gram-negative bacteria were reported [5, 6]. It was demonstrated that several naturally occurring efflux pump inhibitors can restore the activity of antibiotics against MDR bacteria [7, 8]. The present study was therefore designed to investigate the antibacterial potential against MDR Gram-negative phenotypes expressing active efflux pumps of six Cameroonian medicinal plants used traditionally in the treatment of bacterial infections, namely *Anthocleista schweinfurthii* Gilg.(Loganiaceae), *Boehmeria platyphylla* D. Don (Urticaceae), *Caucalis melanantha* (Hochst/Hien) (Urticaceae), *Erigeron floribundus* (H.BK) (Asteraceae), *Nauclea latifolia* Smith (Rubiaceae) and *Zehneria scobra* (cf) Sondev (Cucurbitaceae).

## Methods

### Plant materials and extraction

The plant materials used in this study were collected on April 2013 in West and South West regions of Cameroon and identified by a specialist of the National Herbarium (Table 1). The plants included two trees namely *Anthocleista schweinfurthii* and *Nauclea latifolia*, and four herbs namely *Boehmeria platyphylla, Caucalis melanantha, Erigeron floribundus* and *Zehneria scobra*. The whole plant was collected for herbs whilst leaves, fruits and stem bark were collected for trees. Each plant material was dried at room temperature and powdered using a grinder. One hundred grams of each powder was then macerated in 1 L of pure methanol (MeOH) for 48 h and filtered through Whatman filter paper no.1. The filtrate obtained was concentrated under reduced pressure in a rotary evaporator to obtain the crude extract. All crude extracts were then kept at 4 °C until further uses.

**Table 1:** General informations and report on evidence of biological activities and chemistry of the studied plants

Species (family); Voucher number*	Traditional uses	Parts used traditionally	Area of plant collection	Bioactive or potentially bioactive components	Bioactivities
*Anthocleista schweinfurthii* Gilg.	Hernia, female sterility, stomach-ache in women, ovarian problems, venereal diseases, bronchitis, fever, purgative, malaria, hard abscesses anthelminthic, otitis, ophthalmia, pain, malaria, cancers, venereal diseases, bacterial diseases [21]	Stem bark, roots, Sap of young leaves, leaves	Bagangté, West region of Cameroon	Polyphenols, alkaloids, terpenes and steroids [21], schweinfurthiin 1, bauerenone 2, bauerenol 3, 1-hydroxy-3,7,8 trimethoxy-xanthone 4 and 1, 8-dihydroxy-3, 7 dimethoxy-xanthone 5 [35]	Antibacterial activity against *Staphylococcus aureus* and *Escherichia coli* [21]
(Loganiaceae); 32389/HNC					
*Boehmeria platyphylla* D. Don	Stomachic [36] and dysentery [37], control bleeding [28], skin burns	Roots, leaves	Lebialem, South West region of Cameroon	Acetophenone (3,4-dimethoxy-w-(2'-piperidy1)) [27], cryptopleurine [28].	Nor reported
(Urticaceae); 27550/SRF/CAM					
*Caucalis melanantha* (Hochst/Hien)	Evil eye [38], epilepsy [39], malaria, Stomachaches gastritis [40]	Leaves, roots, whole plant	Lebialem, South West region of Cameroon	α-Pinene, sabinene and terpinen-4-ol [31]	Antifungal activity [31]
(Apiaceae); 32891/HNC					
*Erigeron floribundus* (H.BK)	Skin disorders [32], Acquired immunodefiency syndrome (AIDS) therapy [41], antipyretic, and anti-inflammatory [42], gastrointestinal tract infections	Whole plant	Lebialem, South West region of Cameroon	Saponins, flavonods, tannins, phenols, alkaloids and essential oils [42], Phenolics, olean-3-oleil-12,18 diene [43].	Analgesic and antiinflammatory [42]; antifungal activity against *Epidermophyton floccosum, Microsporum canis, M. gypseum, M. langeronii, Trichophyton mentagrophytes, T. rubrum, T. soudanense* and *Scopulariopsis brevicaulis* [44]
(Asteraceae); 5619S/RF/Cam					
*Nauclea latifolia* Smith	Gonorrhea [45], hypertension [46], gastrointestinal tract disorders [47], prolong menstrual flow [48]. stomach pain, constipation, fever, diarrhoea, piles dysentery [49].	Stem bark, leaves, roots, fruits	Bagangté, West region of Cameroon	Naucleamides A,B,C,D,E [50]	antimicrobial activity of methanol extract against *E. coli, S. dysenteriae, S. aureus, Bacillus subtilis* and *Aspergillus niger* [49]
(Rubiaceae); 34577/HNC					
*Zehneria scobra* (cf) Sondev	Fever, diarrhea, skin diseases, stomach pain, jaundice and kidney infection [51]	Leaves, frits, flowers, roots shoot	Bafou, West region of Cameroon	Gypenoside [51]	Antimicrobial activity of ethanol extract against *E-coli*, *Pseudomonas auruginosa, S. aureus, E. coli* [51], *Vibrio cholerae, Enterobacter aerogenes, Klebsiella pneumoniae*, *Salmonella paratyphi*, *Proteus mirabilis*, *Proteus vulgaris, Bacillus cereus, B. subtilis and Sterptococcus pneumonie* [52]
(Cucurbitaceae); 19668/SRF/CAM					

* HNC Cameroon National Herbarium, *SRF* Société des Réserves Forestières du Cameroun

### Phytochemical screening

The major phytochemical classes such as phenols tannins, flavonoids, saponins, alkaloids, anthraquinones, cardiac glycosides, steroids and triterpenes (Table 2) were investigated according to the common described phytochemical methods [9–13].

**Table 2:** Preliminary chemical composition of the studied plant extracts

Plant	Part used*	Phenols	Tannins	Flavonoids	Saponins	Alkaloids	Anthraquinones	Cardiac glycosides	Steroids	Triterpenes
*A. melanantha*	W	+	+	+	-	-	+	+	-	-
*A. Schweinfurthii*	B	+	+	-	-	-	+	+	-	+
	F	+	+	-	-	-	+	+	-	+
	L	+	+	-	-	-	-	+	-	-
*B. platyphylla*	W	+	+	-	-	-	+	+	-	-
*E. floribundus*	W	+	+	-	-	-	-	+	-	-
*N. latifolia*	B	+	+	-	-	-	+	+	+	+
	F	+	+	-	-	-	+	+	-	+
	L	+	+	-	-	-	+	+	+	+
*Z. scobra*	W	+	-	-	-	+	-	+	-	-

Extract were from [*B* stem bark, *F* fruits, *L* leaves, *W* whole plant]. (+): Present; (-): Absent

### Chemicals for antibacterial assays

Seven commonly used antibiotics including tetracycline (TET), kanamycin (KAN), streptomycin (STR), ciprofloxacin (CIP), norfloxacin (NOR), chloramphenicol (CHL), ampicillin (AMP), erythromycin (ERY) (Sigma-Aldrich, St Quentin Fallavier, France) were used. *p*-Iodonitrotetrazolium chloride 0.2 % (INT) and phenylalanine arginine *β*-naphthylamide (PAβN) (Sigma-Aldrich) were used as bacterial growth indicator and efflux pumps inhibitor respectively.

### Microorganisms and growth conditions

Pathogenic microorganisms used in the present study were Gram-negative bacteria including MDR isolates (Laboratory collection) and reference strains (American Type Culture Collection) of *Escherichia coli* (ATCC8739, ATCC10536, AG100, AG100A, AG100ATet, AG102, MC4100 W3110), *Enterobacter aerogenes* (ATCC13048, CM64, EA27, EA289, EA298, EA294), *Klebsiella pneumoniae* (ATCC11296, KP55, KP63, K24, K2), *Enterobacter cloacae* (ECCI69, BM47, BM67), *Pseudomonas aeruginosa* (PA01, PA124) and *Providencia stuartii* (ATCC29916, NEA16, PS2636, PS299645). The clinical strains were the laboratory collection from UMR-MD1, University of Marseille, France. Their features are reported in Additional file 1: Table S1. They were maintained at 4 °C and sub-cultured on a fresh appropriate Mueller Hinton Agar (MHA) for 24 h before any antibacterial test.

### Antibacterial assays

The MICs of the tested extracts were determined using a rapid INT colorimetric assay [14]. Briefly, test samples were first dissolved in dimethylsulfoxide/ Mueller Hinton Broth (DMSO/MHB). The solution obtained was then added to MHB and serially diluted two fold (in a 96-well microtilter plate). One hundred microliters of inoculums (1.5× 10^6^ CFU/ml) prepared in MHB were then added. The plates were covered, agitated with a shaker to mix the contents of the wells and incubated at 37 °C for 18 h. The final concentration of DMSO was 2.5 %, a concentration at which DMSO does not affect bacterial growth. Wells containing MHB, 100 μl of inoculum, and DMSO at a final concentration of 2.5 % served as the negative control. Chloramphenicol was used as reference antibiotic. The MICs of each extract were detected after 18 h of incubation at 37 °C after addition of 40 μl INT (0.2 mg/ml) and incubation at 37 °C for 30 min. Viable bacteria reduce INT with appearance of a pink dye. The MIC of each sample was defined as its lowest concentration that prevented this change and resulted in the complete inhibition of microbial growth. The Minimum Bactericidal Concentration (MBC) was determined by sub-culturing samples from the wells with concentrations above or equal to the MIC on new plates of Mueller Hinton broth (MHB). The MBC was considered as the lowest concentration of the extract which prevented appearance of pink color after addition of INT. Each assay was performed in triplicate at three different days.

### Antibiotic-modulation assay

To evaluate the antibiotic resistance modifying activity of the extracts, the MIC of antibiotics were determined in the presence or absence of the plant extracts using the broth microdilution technique as described above. After a preliminary assay on two MDR bacteria, *P. aeruginosa* PA124 and *E. aerogenes* CM64 (Additional file 1: Tables S3 and S4), extracts from *A. Schweinfurthii* fruits, *N. Latifolia* leaves and stem bark, and from the whole plant of *Z. scobra* were selected and tested at their MIC/2 and MIC/5 in combination with seven antibiotics (CHL, AMP, KAN, NOR, ERY, TET and STR) on six MDR bacterial strains (*P. aeruginosa* PA124, *E. aerogenes* EA289 and CM64, *E. coli* AG100, *P. stuartii* NAE16 and *K. pneumoniae* K24).

The reverse of Fractional Inhibitory concentration (1/FIC) was calculated as follows:

$$ \mathsf{1}/\mathrm{F}\mathrm{I}\mathrm{C} = \kern0.5em \mathrm{M}\mathrm{I}{\mathrm{C}}_{\mathrm{Antibiotic}\ \mathrm{alone}}/\mathrm{M}\mathrm{I}{\mathrm{C}}_{\mathrm{Antibiotic}\ \mathrm{in}\ \mathrm{combination}\ \mathrm{with}} $$

The interpretation was made as follows: Synergistic (*≥*2), Indifferent (1 to 0.5), or Antagonistic (≤0.25) [5, 15]. All assays were performed in triplicate and repeated thrice.

## Results

### Phytochemical composition of the tested extracts

The main classes of secondary metabolites for each extract were screened and the results are summarized in Table 2. It appears that all the plant extracts of this study possess at least 3 classes of screened secondary metabolites. Only three classes of the screened phytochemicals were detected in the extracts from *Z.* s*cobra, E. floribundus* and *A. schweinfurthii* leaves. Extracts from *N. latifolia* leaves and stem bark contained six phytochemical classes. All the extracts contained phenols and cardiac Glycosides.

### Antibacterial activity

The results (Additional file 1: Table S2) showed that all extracts displayed antibacterial activity against at least 4/28 (14.3 %) tested bacterial strains, with MIC values ranged from 128 to 1024 μg/mL. Extracts from *A. schweinfurthii* leaves, fruits and bark exerted inhibitory effects respectively against 14/28 (50 %), 13/28 (46.4 %) and 8/28 (28.6 %) studied bacteria. The extracts from the fruits and leaves of *N. latifolia* were active respectively on 6/28 (21.4 %) and 7/28 (25 %) tested bacteria whilst the bark extract displayed the best spectrum of activity [active on 22/28 (78.6 %) tested bacteria]. Extracts of *B. platyphylla* and *Erigeron floribundus* also showed large spectra of antibacterial activity with MIC values recorded on 17/28 (60.7 %) and 21/28 (75 %) bacterial strains respectively. *Causalis melanantha* and *Z. scobra* extracts displayed low antibacterial spectra [MIC recorded respectively on 4/28 (14.3 %) and 7/28 (25 %) tested bacterial]. *P. aeuginosa* PA124 appeared to be the most resistant bacteria strain with the sensitivity observed only towards *N. latifolia* bark extract.

### Antibiotic resistance modifying activities of the plant extracts

Preliminary results obtained in two most resistant strains, *P. aeruginosa* PA124 and *E. aerogenes* CM64 (results presented in Additional file 1: Tables S3 and S4) allowed selecting the following extracts: *A. schweinfurthii* fruits, *N. latifolia* leaves and bark and *Z. scobra* as well as the appropriate sub-inhibitory concentrations of MIC/2 and MIC/5 for further studies. From the results summarised in Tables 3, 5, 5 and 6, it appears that all the four extracts improved the activities of antibiotics, from 2 to more than 64 folds. The highest activities were observed with *A. schweinfurthii* fruits (Table 3) and *Z. scobra* (Table 6). *A. schweinfurthii* fruits potentiated the activities of TET on 66.7 % and 50 % of the bacteria strains at MIC/2 and MIC/5 respectively. It also increased the activity of KAN (MIC/2 and MIC/5) and STR (MIC/2) in 50 % of the tested bacterial strains while *Z. scobra* improved the activities of STR on 66.7 % and 50 % of the bacteria strains at MIC/2 and MIC/5 respectively. *Z. scobra* also improved the activity of CHL on 50 % of the tested bacterial strains at the two sub-inhibitory concentrations of MIC/2 and MIC/5 (Table 6). Synergistic effects (50 % of antibiotic activity potentiating at MIC/2 and MIC/5) were observed with *N. Latifolia* leaves extract (Table 5) on TET and STR. The highest rate of improvement of antibiotic activity by *N. Latifolia* stem bark extract was rather noticed on TET and KAN with a rate of 33.3 %. Among the four extracts, this later displayed the lowest antibiotic potentiating effect. Moreover, no synergistic effect was observed with NOR, while synergy between the studied extracts and antibiotics were observed with Ampicilin, with a rate of only 16.67 % (Table 5).

**Table 3:** Effect of sub-inhibitory concentrations of *Anthocleista schweinfurthii* fruits extract on the activities of first line antibiotics against Gram-negative MDR bacteria

Antibiotics and concentrations of extract		Bacterial strains and MIC values (μg/mL)						PBSS (%)
		*K. pneumoniae* K24	*P. stuartii* NAE16	*E. coli* AG100	*E. aerogenes* EA289	*P. aeruginosa* PA124	*E. aerogenes* CM64	
TET	0	64	64	16	8	16	32	-
	MIC/2	64	64(1)^I^	8(2)^S^	8(1)^I^	64(0.25) ^A^	16(2)^S^	33.33
	MIC /5	64	64(1)^I^	8(2)^S^	8(1)^I^	64(0.25)^A^	16(2)^S^	33.33
NOR	0	≥64	1	≤0.25	-	128	2(1)^I^	-
	MIC/2	≥64	1(1)^I^	≤0.25	-	128 (1)^I^	2(1)^I^	0
	MIC/5	≥64	1(1)^I^	≤0.25	-	128 (1)^I^	2(1)^I^	0
STR	0	2	32	≥256	64	64	16	-
	MIC/2	2(1)^I^	16(2)^S^	≥256	8(8)^S^	32(2)^S^	16(1)^I^	50
	MIC/5	2(1)^I^	32(1)^I^	≥256	8(8)^S^	32(2)^S^	16(1)^I^	33.33
KAN	0	8	8	8	32	64	≤2	-
	MIC/2	8(1)^I^	8(1)^I^	2(4)^S^	2(16)^S^	32(2)^S^	≤2	50
	MIC/5	8(1)^I^	8(1)^I^	4(2)^S^	2(16)^S^	32(2)^S^	≤2	50
CHL	0	16	8	8	512	64	512	-
	MIC/2	8(2)^S^	(2)^S^	8(1)^I^	256(2)^S^	64(1)^I^	256(2)^S^	66.67
	MIC/5	8(2)^S^	(2)^S^	8(1)^I^	512(1)^I^	64(1)^I^	256(2)^S^	50
ERY	0	128	16(1)^I^	64	128	128	256	-
	MIC/2	64(2)^S^	16(1)^I^	64(1)^I^	128(1)^I^	128(1)^I^	128(2)^S^	33.33
	MIC/5	128(1)^I^	16(1)^I^	64(1)^I^	128(1)^I^	128(1)^I^	128(2)^S^	16.67
AMP	0	-	-	128	-	-	-	-
	MIC/2	-	-	32(4)^S^	-	-	-	16.67
	MIC/5	-	-	128(1)^I^	-	-	-	0

(-) : >256 μg/ml; (): fold decrease in MIC values of the antibiotics after association with plants extract; S: Synergy, I: Indifference, A: antagonism, Antibotics (*CHL* chloramphenicol, *AMP* ampicillin, *KAN* kanamycin, *NOR* norfloxacin, *ERY* erythromycin, *TET* tetracycline, *STR* streptomycin); *PBSS* percentage of bacteria strain on which synergism has been observed

## Discussion

Medicinal plants are potential source of antimicrobial agents used in the treatment of infectious diseases [16]. According to Rios and Recio [17], and Kuete et al. [17], the antibacterial activity of a plant extract is considered significant when the MICs are below 100 μg/mL. The activity is considered moderate when 100 ≤ MIC ≤ 625 μg/mL and weak when MIC are above 625 μg/mL [17]. Therefore, the antibacterial activities reported in the present study can mostly be regarded as moderate or low. This could be explained by the fact that the tested bacteria are mostly MDR phenotypes. In fact, *P. aeruginosa* and MDR Enterobacteriaceae ( *K. pneumoniae, E. aerogenes, E.cloacae* and *P. stuartii* and *E. coli*) tested in the present study have been classified as antimicrobial-resistant organisms of concern in healthcare facilities [18–20]. The previously reported activities of *A. schweinfurthii* include antibacterial inhibitory effects of n-hexane, dichloromethane, ethyl acetate and methanol extracts from leaves and stem bark against *Staphylococcus aureus* ATCC 33591 and *E. coli* ATCC 27195 [21]. The MIC values obtained in the present study were respectively 62.5 and 125 μg/ml against *S. aureus* and *E. coli*. Such values were higher than those previously documented, highlighting the MDR feature of the studied bacteria. MBC values were obtained in few cases (Additional file 1: Table S2). A keen look of data (Additional file 1: Table S2) indicates that, in most of the cases, the tested extract exerted bacteriostatic effects with a ratio MBC/MIC above 4. The overall antibacterial activity of the tested extracts could be due their phytochemical composition. However, the presence of a specific class of second metabolite could not guarantee the antibacterial activity of the plant, as this will depend on nature of the compounds, its concentration as well as the possible interactions with other constituents of the extract. It is also surprising that saponins, known to possess antibacterial activities were not detected in the tested extracts; However, this does means that the extract were completely devoid of this class of secondary metabolite; One of the most understandable explanation should that saponins could be present in very little amounts in the tested extract, and therefore could not be detected using the qualitative phytochemical methods. Some cardiac glycosides such as bufalin, oubain, digoxin are toxic meanwhile many of them have therapeutic uses and these primarily involve the treatment of cardiac failure [22–24]. Their utility results from an increased cardiac outpout by increasing the force of contraction. By increasing intracellular calcium, cardiac glycosides increase calcium-induced calcium release and thus contraction [23, 24]. The traditional use of the studied plants could suggest that their cardiac glycoside could be not toxic and have very low toxic effects.

To the best of our knowledge, the present work describes for the first time the antibacterial activity of *B. platyphylla.* This activity could be due to the presence of the detected phytochemicals. In fact, antibacterial compounds such as acetophenone [25] and cryptopleurine [26–28] were previously isolated from *B. Platyphylla*. The antibacterial activity of *C. melanantha* and *E. floribundus* is also reported here for the first time. However these plants were previously reported for their antifungal activities [29–32]. The antibacterial activities of extracts from *Zehneria scobra* and *Nauclea latifolia* [33, 34] were reported on some bacteria: The present study therefore provides additional information on the activity of these plants against MDR Gram-negative phenotypes.

The synergistic effects between antibiotics and the tested plants are also reported here for the first time. The observed synergistic effects could be due to possible interaction between plant constituents and the tested antibiotics. As the strains used in this study are known to actively expressed efflux pumps, one of the possible explanations for the observed synergistic effects could be the ability of the constituents of the extracts to act as efflux pumps inhibitor. This can explain why the effect of antibiotics with intracellular targets such as STR, CHL and KAN increased contrary to that of beta-lactamine (AMP) acting in the cell wall (Tables 3, 4, 5 and 6).

**Table 4:** Effect of sub-inhibitory concentrations of *Nauclea latifolia* stem bark extract on the activities of first line antibiotics against Gram-negative MDR bacteria

Antibiotics and concentrations of extract		Bacterial strains and MIC values (μg/mL)						PBSS (%)
		*K. pneumoniae* K24	*P. stuartii* NAE16	*E. coli* AG100	*E. aerogenes* EA289	*P. aeruginosa* PA124	*E. aerogenes* CM64	
TET	0	64	64	16	8	16	32	-
	MIC/2	64(1)^I^	64(1)^I^	8(2)^S^	8(1)^I^	16(1)^I^	16(2)^S^	33.33
	MIC /5	64(1)^I^	64(1)^I^	16(1)^I^	8(1)^I^	16(1)^I^	16(2)^S^	16.67
NOR	0	≥64	1	≤0.25	-	128	2(1)^I^	-
	MIC/2	≥64	1(1)^I^	≤0.25	-	128 (1)^I^	2(1)^I^	0
	MIC /5	≥64	1(1)^I^	≤0.25	-	128(1)^I^	2(1)^I^	0
STR	0	2	32	≥256	64	64	16	-
	MIC/2	2(1)^I^	32(1)^I^	≥256	32(2)^S^	64(1)^I^	8(2)^S^	16.67
	MIC /5	2(1)^I^	32(1)^I^	≥256	64(1)^I^	64(1)^I^	16(1)^I^	0
KAN	0	8	8	8	32	64	≤2	-
	MIC/2	8(1)^I^	8(1)^I^	4(2)^S^	16(2)^S^	64(1)^I^	≤2	33.33
	MIC /5	8(1)^I^	8(1)^I^	8(1)^I^	32(1)^I^	64(1)^I^	≤2	16.67
CHL	0	16	8	8	512	64	512	-
	MIC/2	16(1)^I^	4(2)^S^	8(1)^I^	512(1)^I^	64(1)^I^	512	16.67
	MIC /5	16(1)^I^	8(1)^I^	8(1)^I^	512(1)^I^	64(1)^I^	512	0
ERY	0	128	16(1)^I^	64	128	128	256	-
	MIC/2	128(1)^I^	16(1)^I^	64(1)^I^	128(1)^I^	128(1)^I^	128(2)^S^	16.67
	MIC /5	128(1)^I^	16(1)^I^	64(1)^I^	128(1)^I^	128(1)^I^	256(1)^I^	0
AMP	0	-	-	128	-	-	-	-
	MIC/2	-	-	256(2)^I^	-	-	-	0
	MIC /5	-	-	256(2)^I^	-	-	-	0

(-) : >256 μg/ml; (): fold decrease in MIC values of the antibiotics after association with plants extract; S: Synergy, I: Indifference; A: antagonism, Antibotics (*CHL* chloramphenicol, *AMP* ampicillin, *KAN* kanamycin, *NOR* norfloxacin, *ERY* erythromycin, *TET* tetracycline, *STR* streptomycin); *PBSS* percentage of bacteria strain on which synergism has been observed

**Table 5:** Effect of sub-inhibitory concentrations of *Nauclea latifolia* leaves extract on the activities of first line antibiotics against Gram-negative MDR bacteria

Antibiotics and concentrations of extract		Bacterial strains and MIC values (μg/mL)						PBSS (%)
		*K. pneumoniae* K24	*P. stuartii* NAE16	*E. coli* AG100	*E. aerogenes* EA289	*P. aeruginosa* PA124	*E. aerogenes* CM64	
TET	0	64	64	16	8	16	32	-
	MIC/2	64(1)^I^	32(2)^S^	8(2)^S^	2(4)^S^	16(1)^I^	32(1)^I^	50.00
	MIC /5	64(1)^I^	32(2)^S^	8(2)^S^	4(2)^S^	16(1)^I^	32(1)^I^	50.00
NOR	0	≥64	1	≤0.25	-	128	2(1)^I^	-
	MIC/2	≥64	1(1)^I^	≤0.25	-	128 (1)^I^	2(1)^I^	0
	MIC /5	≥64	1(1)^I^	≤0.25	-	128 (1)^I^	2(1)^I^	0
STR	0	2	32	≥256	64	64	16	-
	MIC/2	2(1)^I^	16(2)^S^	32(≥8)^S^	16(4)^S^	64(1)^I^	2(8)^S^	50.00
	MIC /5	2(1)^I^	32(1)^I^	32(≥8)^S^	32(2)^S^	64(1)^I^	8(2)^S^	50.00
KAN	0	8	8	8	32	64	≤2	
	MIC/2	8(1)^I^	8(1)^I^	2(4)^S^	16(2)^S^	64(1)^I^	≤2	33.33
	MIC /5	8(1)^I^	8(1)^I^	4(2)^S^	32(1)^I^	64(1)^I^	≤2	16.67
CHL	0	16	8	8	512	64	512	-
	MIC/2	16(1)^I^	4(2)^S^	8(1)^I^	256(2)^S^	64(1)^I^	512	33.33
	MIC /5	16(1)^I^	4(2)^S^	8(1)^I^	256(2)^S^	64(1)^I^	512	33.33
ERY	0	128	16(1)^I^	64	128	128	256	-
	MIC/2	128(1)^I^	16(1)^I^	64(1)^I^	128(1)^I^	128(1)^I^	256(1)^I^	0
	MIC /5	128(1)^I^	16(1)^I^	64(1)^I^	128(1)^I^	128(1)^I^	256(1)^I^	0
AMP	0	-	-	128	-	-	-	-
	MIC/2	-	-	64(2)^S^	-	-	-	16.67
	MIC /5	-	-	128(1)^I^	-	-	-	0

(-) : >256 μg/ml; (): fold decrease in MIC values of the antibiotics after association with plants extract; S: Synergy, I: Indifference; A: antagonism, Antibotics (*CHL* chloramphenicol, *AMP* ampicillin, *KAN* kanamycin, *NOR* norfloxacin, *ERY* erythromycin, *TET* tetracycline, *STR* streptomycin); *PBSS* percentage of bacteria strain on which synergism has been observed

**Table 6:** Effect of sub-inhibitory concentrations of *Zehneria scobra* extract on the activities of first line antibiotics against Gram-negative MDR bacteria

Antibiotics and concentrations of extract		Bacterial strains and MIC values (μg/mL)						PBSS (%)
		*K. pneumoniae* K24	*P. stuartii* NAE16	*E. coli* AG100	*E. aerogenes* EA289	*P. aeruginosa* PA124	*E. aerogenes* CM64	
TET	0	64	64	16	8	16	32	-
	MIC/2	64(1)^I^	64(1)^I^	8(2)^S^	8(1)^I^	16(1)^I^	32(1)^I^	16.67
	MIC/5	64(1)^I^	64(1)^I^	8(2)^S^	8(1)^I^	16(1)^I^	32(1)^I^	16.67
NOR	0	≥64	1	≤0.25	-	128	2(1)^I^	-
	MIC/2	≥64	1(1)^I^	≤0.25	-	128 (1)^I^	2(1)^I^	0
	MIC/5	≥64	1(1)^I^	≤0.25	-	128 (1)^I^	2(1)^I^	0
STR	0	2	32	≥256	64	64	16	-
	MIC/2	2(1)^I^	16(2)^S^	≥256	8(8)^S^	32(2)^S^	4(4)^S^	66.67
	MIC/5	2(1)^I^	16(2)^S^	≥256	16(4)^S^	32(2)^S^	4(4)^S^	50
KAN	0	8	8	8	32	64	≤2	-
	MIC/2	8(1)^I^	8(1)^I^	4(2)^S^	4(8)^S^	64(1)^I^	≤2	33.33
	MIC/5	8(1)^I^	8(1)^I^	8(1)^I^	8(4)^S^	64(1)^I^	≤2	16.67
CHL	0	16	8	8	512	64	512	-
	MIC/2	8(2)^S^	(2)^S^	8(1)^I^	512(1)^I^	64(1)^I^	256(2)^S^	50
	MIC/5	8(2)^S^	8(1)^I^	8(1)^I^	512(1)^I^	64(1)^I^	256(2)^S^	50
ERY	0	128	16(1)^I^	64	128	128	256	-
	MIC/2	128(1)^I^	16(1)^I^	64(1)^I^	128(1)^I^	128(1)^I^	128(2)^S^	16.67
	MIC/5	128(1)^I^	16(1)^I^	64(1)^I^	128(1)^I^	128(1)^I^	256(1)^I^	0
AMP	0	-	-	128	-	-	-	-
	MIC/2	-	-	128(1)^I^	-	-	-	0
	MIC/5	-	-	128(1)^I^	-	-	-	0

(-) : >256 μg/ml; (): fold decrease in MIC values of the antibiotics after association with plants extract; S: Synergy. I: Indifference; A: antagonism. Antibotics (*CHL* chloramphenicol, *AMP* ampicillin, *KAN* kanamycin, *NOR* norfloxacin, *ERY* erythromycin, *TET* tetracycline, *STR* streptomycin); *PBSS* percentage of bacteria strain on which synergism has been observed

## Conclusion

The overall results of the present study provides baseline information for the possible use of the tested plants, especially *A. schweinfurthii*, *N. Latifolia, B. platyphylla* and *E. floribundus* in the control of infections due to Gram-negative bacteria. The present study indicates that the tested plant extracts alone could not be used efficiently to tackle MDR bacterial infections. However, it was demonstrated that extracts from *A. schweinfurthii* fruits and *Z. scobra* could be used in combination with some antibiotics to fight bacterial multi-drug resistance.

### Availability of data and materials

The datasets supporting the conclusions of this article are presented in this main paper and supporting material. Plant materials used in this study have been identified at the Cameroon National Herbarium where voucher specimens are deposited.

### Consent for publication

Not applicable in this section.

### Ethic approval and consent to participate

Not applicable in this section.

## Additional file

Additional file 1: Table S1. Bacterial strains and features. **Table S2.** Minimal inhibitory concentration (MIC) and minimal bactericidal (MBC) of the plant extracts and CHL on the studied bacteria. **Table S3.** Effects of different concentrations of plant extracts on the MIC (μg/ml) of antibiotics against *P. aeruginosa* PA124. **Table S4.** Effects of different concentrations of plant extracts on the MIC (μg/ml) of antibiotics against *E. aerogenes* CM64 (DOC 320 kb)

## Abbreviations

*A schweinfurthii*: *Anthocleista schweinfurthii*
AMP: ampicillin
ATCC: American type culture collection
*B. platyphylla*: *Boehmeria platyphylla*
*C melanantha*: *Caucalis melanantha*
CFU: colony forming unit
CHL: chloramphenicol
CIP: ciprofloxacin
DMSO: dimethylsulfoxide
*E floribundus*: *Erigeron floribundus*
*E. aerogenes*: *Enterobacter aerogenes*
*E. cloacae*: *Enterobacter cloacae*
*E. coli*: *Escherchia coli*
ERY: erythromycin
FIC: fractional inhibitory concentration
INT: *p*-iodonitrotetrazolium chloride
*K. pneumoniae*: *Klebsiella pneumoniae*
KAN: kanamycin
MBC: minimum bactericidal concentration
MDR: multi-drug resistant
MeOH: methanol
MHA: Mueller Hinton agar
MHB: Mueller Hinton broth
MIC: minimum inhibitory concentration
*N. latifolia*: *Nauclea latifolia*
NOR: norfloxacin
*P. aeruginosa*: *Pseudomona aeruginosa*
*P. stuartii*: *Providencia stuartii*
PAβN: phenylalanine arginine *β*-naphthylamide
STR: streptomycin
TET: tetracycline
*Z scobra*: *Zehneria scobra*

## References

[1] Walsh TR, Toleman MA, Poirel L, Nordmann P (2005). Metallo-β-lactamase: the quiet before the storm. Clin Microbiol Rev.

[2] Targant H. L’îlot de multirésistance aux antibiotiques, *Salmonella* Genomic Island 1 (SGI1): variabilité, diffusion inter - espèces et implication dans la virulence. Lyon: Université Claude Bernard de Lyon 1; 2010.

[3] Poole K (2004). Efflux-mediated multiresistance in Gram-negative bacteria. Clin Microbiol Infect.

[4] Kuete V (2010). Potential of Cameroonian plants and derived products against microbial infections: a review. Planta Med.

[5] Noumedem J, Mihasan M, Kuiate J, Stefan M, Cojocaru D, Dzoyem J, Kuete V. *In vitro* antibacterial and antibiotic-potentiation activities of four edible plants against multidrug-resistant Gram-negative species. BMC Complement Altern Med. 2013; 13:190. 10.1186/1472-6882-13-190 PMC3734210 23885762

[6] Lacmata ST, Kuete V, Dzoyem JP, Tankeo SB, Teke GN, Kuiate JR, Pages J-M (2012). Antibacterial activities of selected Cameroonian plants and their synergistic effects with antibiotics against bacteria expressing MDR phenotypes. Evid Based Complement Alternat Med.

[7] Nelson ML, Levy SB (1999). Reversal of tetracycline resistance mediated by different bacterial tetracycline resistance determinants by an inhibitor of the Tet(B) antiport protein. Antimicrob Agents Chemother.

[8] German N, Wei P, Kaatz GW, Kerns RJ (2008). Synthesis and evaluation of fluoroquinolone derivatives as substrate-based inhibitors of bacterial efflux pumps. Eur J Med Chem.

[9] Harbone JB (1973). Phytochemical methods: A guide to modern techniques of plant analysis.

[10] Harbone J (ed.). Phytochemical methods: a guide to modern techniques of plant analysis. London: Chapman & Hall; 1973.

[11] Ngameni B, Fotso GW, Kamga J, Ambassa P, Abdou T, Fankam AG, Voukeng IK, Ngadjui BT, Abegaz BM, Kuete V, Kuete V (2013). 9 - Flavonoids and Related Compounds from the Medicinal Plants of Africa. Medicinal Plant Research in Africa.

[12] Wansi JD, Devkota KP, Tshikalange E, Kuete V, Kuete V (2013). 14 - Alkaloids from the Medicinal Plants of Africa. Medicinal Plant Research in Africa.

[13] Poumale HMP, Hamm R, Zang Y, Shiono Y, Kuete V, Kuete V (2013). 8 - Coumarins and Related Compounds from the Medicinal Plants of Africa. Medicinal Plant Research in Africa.

[14] Eloff JN (1998). A sensitive and quick microplate method to determine the minimal inhibitory concentration of plant extracts for bacteria. Planta Med.

[15] Coutinho HD, Lima JG, Siqueira-Júnior JP (2010). Additive effects of *Hyptis martiusii* Benth with aminoglycosides against *Escherichia coli*. Indian J Med Res.

[16] Rios J, Recio M (2005). Medicinal plants and antimicrobial activity. J Ethnopharmacol.

[17] Kuete V, Kuete V (2013). Medicinal Plant Research in Africa. Pharmacology and Chemistry.

[18] Tran QT, Mahendran KR, Hajjar E, Ceccarelli M, Davin-Regli A, Winterhalter M, Weingart H, Pages JM (2010). Implication of porins in beta-lactam resistance of *Providencia stuartii*. J Biol Chem.

[19] Kuete V, Ngameni B, Tangmouo JG, Bolla JM, Alibert-Franco S, Ngadjui BT, Pages JM (2010). Efflux pumps are involved in the defense of Gram-negative bacteria against the natural products isobavachalcone and diospyrone. Antimicrob Agents Chemother.

[20] Kuete V, Alibert-Franco S, Eyong KO, Ngameni B, Folefoc GN, Nguemeving JR, Tangmouo JG, Fotso GW, Komguem J, Ouahouo BM, Bolla JM, Chevalier J, Ngadjui BT, Nkengfack AE, Pagès JM (2011). Antibacterial activity of some natural products against bacteria expressing a multidrug-resistant phenotype. Int J Antimicrob Agents.

[21] Ngbolua K-t-N, Mubindukila REN, Mpiana PT, Ashande MC, Baholy R, Ekutsu GE, Gbolo ZB, Fatiany PR. *In vitro* assessment of antibacterial and antioxidant activities of a Congolese medicinal plant species *Anthocleista schweinfurthii* Gilg (Gentianaceae). J Modern Drug Discov Drug Deliv Res. 2014;3:1-6.

[22] Mbaveng AT, Hamm R, Kuete V. 19 - Harmful and Protective Effects of Terpenoids from African Medicinal Plants. In: Kuete V, editor. Toxicological Survey of African Medicinal Plants. Oxford: Elsevier; 2014. p. 557-576.

[23] Cowan MM (1999). Plant products as antimicrobial agents. Clin Microbiol Rev.

[24] Bruneton J (1999). Toxic plants. Dangerous to humans and animals.

[25] Al-Shamma A, Drake SD, Guagliardi LE, Mitscher LA, Swayze JK (1982). Antimicrobial alkaloids from *Boehmeria cylindrica*. Phytochemistry.

[26] Choudhary AN, Juyal V (2011). Synthesis of chalcone and their derivatives as antimicrobial agents. Int J Pharm Pharmaceut Sci.

[27] Hart N, Johns S, Lamberton J (1968). 3,4-Dimethoxy-ω-(2'-piperidyl)acetophenone, a new alkaloid from *Boehmeria platyphylla* Don. (family Urticaceae). Aust J Chem.

[28] Bhattarai KR, Maren IE, Chaudhary RP (2011). Medicinal plant knowledge of the Panchase region in the middle hills of the Nepalese Himalayas. Banko Janakari.

[29] Kuiate J, Kuate S, Kemadjou N, Djokoua S, Zifack F, Foko J (2006). Antidermatophitic activities of nine (9) essential oils. East Centr Afr J Pharmaceut Sci.

[30] Kuiate J-R, Tsona AA, Foko J, Bessiere JM, Menut C, Zollo P-HA (2005). Chemical composition and *in vitro* antifungal properties of essential oils from leaves and flowers of *Erigeron floribundus* (H.B. et K.) Sch. Bip. From Cameroon. J Essent Oil Res.

[31] Tchoumbougnang F, Jazet DPM, Wouatsa NAV, Fekam BF, Sameza ML, Amvam ZPH, Menut C (2013). Composition and antifungal properties of essential oils from five plants growing in the mountainous area of the West Cameroon. J Essent Oil Bear Plants.

[32] Bi FT, Koné M, Kouamé N (2008). Antifungal activity of *Erigeron floribundus* (Asteraceae) from Cote d´Ivoire, West Africa. Trop J Pharmaceut Res.

[33] Akoachere J-FTK, Suylika Y, Mbah AJ, Ayimele AG, Assob JCN, Chegaing Fodouop SP, Kodjio N, Gatsing D (2015). *In vitro* antimicrobial activity of agents from *Spilanthes filicaulis* and *Laportea ovalifolia* against some drug resistant bacteria. Br J Pharmaceut Res.

[34] Okwulehie IC, Akanwa FE (2013). Antimicrobial activity of ethanol extract of four indigenous plants from South Eastern Nigeria. ARPN J Sci Technol.

[35] Mbouangouere R, Tane P, Ngamga D, Khan S, Choudhary M, Ngadjui B (2007). A New Steroid and Î-glucosidase Inhibitors from *Anthocleista schweinfurthii*. Res J Med Plant.

[36] Rajbhandari KR. Ethnobotany of Nepal, 1st edition edn. Kathmandu: Ethnobotanical Society of Nepal; 2001.

[37] Narayan EM (2002). Plants and people of Nepal. Narayan E Manandhar.

[38] Kidane B, Andel T, Maesen LJG, Asfaw Z (2014). Use and management of traditional medicinal plants by male and Ari ethnic communities in southern Ethiopia. J Ethnobiol Ethnomed.

[39] Yineger H, Kelbess E, Bekel T, Luleka E (2007). Ethnoveterinary medicinal plants at Bale Mountains National Park, Ethiopia. J Ethnopharmacol.

[40] Focho DA, Ndam WT, Fonge BA (2009). Medicinal plants of Aguambu – Bamumbu in the Lebialem highlands, southwest province of Cameroon. Afr J Pharm Pharmacol.

[41] Yapo F, Yapi F, Ahiboh H, Hauhouot-Attounbre M-L, Guédé N, Djaman J, Monnet D (2011). Immunomodulatory effect of the aqueous extract of *Erigeron floribundus* (Kunth) Sch Beep (Asteraceae) Leaf in Rabbits. Trop J Pharmaceut Res.

[42] Asongalem E, Foyet H, Ngogang J, Folefoc G, Dimo T, Kamtchouing P (2004). Analgesic and antiinflammatory activities of *Erigeron floribundus*. J Ethnopharmacol.

[43] Berto C, Maggi F, Nya P, Pettena A, Boschiero I, Dall'Acqua S (2014). Phenolic constituents of *Erigeron floribundus* (Asteraceae), a Cameroonian medicinal plant. Nat Prod Commun.

[44] Tra Bi F, Koné M, Kouamé N (2008). Antifungal activity of *Erigeron floribundus* (Asteraceae) from Côte d’Ivoire, West Africa. Trop J Pharmaceut Res.

[45] Abbiw DK. Useful plants of Ghana: West African uses of wild and cultivated plants. London: Intermediate Technology Publications; 1990.

[46] Akabue P, Mittal H (1982). Clinical evaluation of a traditional herbal practice in Nigeria: A preliminary report. J Ethnopharmacol.

[47] Madubunyi I (1995). Anti- hepatotoxic and trypanocidal activities of the ethanolic extract of *Nauclea latifolia* root bark. J Herbs Spices Med Plants.

[48] Elujoba A (1995). Female infertility in the hands of traditional birth attendants in South-West Nigeria. Fitoterapia.

[49] Anowi CF, Nnabuife CC, Mbah C, Onyekaba T (2012). Antimicrobial properties of the methanolic extract of the leaves of *Nauclea latifolia*. Int J Drug Res Technol.

[50] Shigemori H, Kagata T, Ishiyama H, Morah F, Ohsaki A, Kobayashi J (2003). Naucleamides A-E: new monoterpene indole alkaloids from *Nauclea latifolia*. Chem Pharmaceut Bull.

[51] Tegenu G (2011). Antimicrobial activity of solvent extracts of *Cucumis ficifolius* and *Zehneria scabra* on test microorganisms.

[52] Arulappan MT, Britto JS, Ruckmani K, Kumar MR (2015). Antimicrobial and antifungal activities of *Zehneria scabra* (L.F.) sond against human pathogens. Int J Develop Res.

