# Supplementary material for: Antibacterial and antibiotic-modulation activity of six Cameroonian medicinal plants against Gram-negative multi-drug resistant phenotypes
Source: BMC Complement Altern Med. 2016 May 4;16:124. doi: 10.1186/s12906-016-1105-1 (PMC4855409; doi:10.1186/s12906-016-1105-1)
Supplement: Additional file 1: Table S1. — Bacterial strains and features. Table S2. Minimal inhibitory concentration (MIC) and minimal bactericidal (MBC) of the plant extracts and CHL on the studied bacteria. Table S3. Effects of different concentrations of plant extracts on the MIC (μg/ml) of antibiotics against P. aeruginosa PA124. Table S4. Effects of different concentrations of plant extracts on the MIC (μg/ml) of antibiotics against E. aerogenes CM64 (DOC 320 kb) [file 12906_2016_1105_MOESM1_ESM.doc]

**Antibacterial and Antibiotic-modulation Activity of Six Cameroonian Medicinal Plants against Gram-negative Multi-drug Resistant Phenotypes**

**Doriane E. Djeussi ^a^, Jaurès A.K. Noumedem^a^,** Bonaventure T. Ngadjui^b^ **and Victor Kuete^a^**

*^a^Department of Biochemistry, Faculty of science, University of Dschang, Cameroon*

*^b^Department of Organic Chemistry, Faculty of Science, University of Yaoundé I, Yaoundé, Cameroon*

**Corresponding author:*

*Tel : +237 677 35 59 27 ; Fax: +237 242 22 60 18. P.O. Box 67 Dschang, Cameroon; E-mail: kuetevictor@yahoo.fr (Prof. Dr. Victor Kuete)*

**[See Main Manuscript]**

**Table S1.** Bacterial strains and features

| **Strains** | **Features** | **References** |
| --- | --- | --- |
| ***Escherichia coli*** |  |  |
| ATCC8739 and ATCC10536 | Reference strains |  |
| AG100 | Wild-type *E. coli* K-12 | [1] |
| AG100A | AG100 *ΔacrAB*::KAN^R^ | [1, 2] |
| AG100A_TET_ | Δ*acrAB* mutant AG100, owing *acrF* gene markedly over-expressed; TET^R^ | [1] |
| AG102 | Δ*acrAB* mutant AG100 | [3] |
| MC4100 | Wild type *E. coli* |  |
| W3110 | Wild type *E. coli* | [4, 5] |
| ***Enterobacter aerogenes*** |  |  |
| ATCC13048 | Reference strains |  |
| EA-CM64 | CHL^R^ resistant variant obtained from ATCC13048 over-expressing the AcrAB pump | [6] |
| EA27 | Clinical MDR isolate exhibiting energy-dependent norfloxacin and chloramphenicol efflux with KAN^R^ AMP^R^ NAL^R^ STR^R^ TET^R^ | [7] |
| EA289 | KAN sensitive derivative of EA27 | [8] |
| EA298 | EA 289 *tolC::*KAN^R^ | [8] |
| EA294 | EA 289 *ΔacrAB*: *::*KAN^R^ | [8] |
| ***Enterobacter cloacae*** |  |  |
| ECCI69 | Clinical isolates | Laboratory collection of UNR-MD1, University of Marseille, France |
| BM47 | Clinical isolates | Laboratory collection of UNR-MD1, University of Marseille, France |
| BM67 | Clinical isolates | Laboratory collection of UNR-MD1, University of Marseille, France |
| ***Klebsiella pneumoniae*** |  |  |
| ATCC12296 | Reference strains |  |
| KP55 | Clinical MDR isolate, TET^R^ , AMP^R^, ATM^R^, CEF^R^ | [9] |
| KP63 | Clinical MDR isolate, TET^R^, CHL^R^, AMP^R^, ATM^R^ | [9] |
| K24 | AcrAB-TolC | Laboratory collection of UNR-MD1, University of Marseille, France |
| K2 | AcrAB-TolC | Laboratory collection of UNR-MD1, University of Marseille, France |
| ***Providencia stuartii*** |  | **[10]** |
| NEA16 | Clinical MDR isolate, AcrAB-TolC |
| ATCC29914 | Clinical MDR isolate, AcrAB-TolC |
| PS2636 | Clinical MDR isolate, AcrAB-TolC |
| PS299645 | Clinical MDR isolate, AcrAB-TolC |
| ***Pseudemonas aeruginosa*** |  |  |
| PA 01 | Reference strains |  |
| PA 124 | MDR clinical isolate | [11] |

^a^AMP, ATM^R^, CEF^R^, CFT^R^, CHL^R^, FEP^R^, KAN^R^, MOX^R^, STR^R^, TET^R^. Resistance to ampicillin, aztreonam, cephalothin, cefadroxil, chloramphenicol, cefepime, kanamycin, moxalactam, streptomycin, and tetracycline; MDR : Multidrug resistant.

Table S2. Minimal inhibitory concentration (MIC) and minimal bactericidal (MBC) of the plant extracts and CHL on the studied bacteria

| **Bacterial strains** | **Tested samples, MIC and MBC (in bracket) in µg/mL** | | | | | | | | | | |
| --- | --- | --- | --- | --- | --- | --- | --- | --- | --- | --- | --- |
|  | ***A. schweinfurthii*** | | | ***B. platyphylla*** | ***C. melanantha*** | ***E. floribondus*** | ***N. latifolia*** | | | *Z. scobra* | Chloramphenicol |
|  | **L** | **F** | **B** | **W** | **W** | **W** | **L** | **F** | **B** | **W** |  |
| ***Escherichia coli*** |  |  |  |  |  |  |  |  |  |  |  |
| ATCC8739 | - (-) | 1024 (-) | - (-) | 1024 (-) | 1024(-) | 512(-) | -(-) | 1024(-) | 1024(-) | 1024(-) | 4(64) |
| ATCC10536 | - (-) | - (-) | 1024 (-) | 512 (-) | 1024(-) | 512(-) | -(-) | -(-) | 1024(-) | 1024(-) | 2(64) |
| AG100 | 1024 (-) | - (-) | - (-) | - (-) | -(-) | 512(-) | -(-) | -(-) | 1024(-) | -(-) | 8(-) |
| AG100A | 512 (-) | 1024 (-) | 1024 (-) | 256 (1024) | -(-) | 1024(1024) | 1024(-) | -(-) | -(-) | -(-) | 4(-) |
| AG100A_TET_ | - (-) | - (-) | - (-) | 1024 (-) | -(-) | 512(-) | -(-) | 1024(-) | 512(1024) | 1024(-) | 32(-) |
| AG102 | - (-) | - (-) | - (-) | 1024(1024) | -(-) | 1024(-) | -(-) | 1024(1024) | 512(-) | -(-) | 8(-) |
| MC4100 | - (-) | 1024 (-) | - (-) | 1024(1024) | -(-) | 1024(1024) | -(-) | -(-) | 1024(1024) | -(-) | 32(-) |
| W3110 | 512 (-) | 1024 (-) | - (-) | 1024(1024) | -(-) | 1024(1024) | -(-) | -(-) | -(-) | 1024(1024) | 8 |
| ***Enterobacter aerogenes*** |  |  |  |  |  |  |  |  |  |  |  |
| ATCC13048 | 256 (-) | 1024 (-) | 256 (-) | 1024 (-) | -(-) | 1024(-) | -(-) | -(-) | 512(-) | -(-) | 16(128) |
| CM64 | - (-) | - (-) | - (-) | - (-) | -(-) | 1024(-) | -(-) | -(-) | 1024(-) | -(-) | 512(-) |
| EA27 | 1024 (-) | - (-) | - (-) | 1024(1024) | -(-) | 1024(1024) | -(-) | -(-) | 1024(-) | 1024(-) | 128(-) |
| EA289 | - (-) | - (-) | - (-) | 1024 (-) | -(-) | -(-) | 1024(-) | -(-) | 512(-) | -(-) | 512(-) |
| EA298 | 512 (-) | 1024 (-) | 1024 (-) | 512 (-) | -(-) | 512(-) | 1024(1024) | 1024(-) | 512(-) | 1024(1024) | 256(-) |
| EA294 | 1024 (-) | 1024 (-) | - (-) | 1024(1024) | 1024(-) | 512(512) | -(-) | 1024(1024) | 512(1024) | -(-) | 4(32) |
| ***Enterobacter cloacae*** |  |  |  |  |  |  |  |  |  |  |  |
| ECCI69 | 1024 (-) | 512 (-) | - (-) | 512 (-) | -(-) | - | -(-) | -(-) | -(-) | -(-) | 256(-) |
| BM47 | - (-) | - (-) | - (-) | - (-) | -(-) | 1024(1024) | -(-) | -(-) | 1024(-) | -(-) | 512(-) |
| BM67 | 512 (-) | - (-) | 1024 (-) | - (-) | -(-) | -(-) | -(-) | -(-) | -(-) | -(-) | 256(-) |
| ***Klebsiella pneumoniae*** |  |  |  |  |  |  |  |  |  |  |  |
| ATCC11296 | 128 (-) | 256 (-) | - (-) | - (-) | -(-) | -(-) | -(-) | -(-) | 1024(1024) | -(-) | 16(128) |
| KP55 | - (-) | - (-) | - (-) | 1024(1024) | -(-) | -(-) | 1024(-) | -(-) | 1024(1024) | -(-) | 64(256) |
| KP63 | - (-) | - (-) | - (-) | - (-) | 1024(-) | 512(-) | 512(-) | 1024(-) | 512(-) | -(-) | 128(-) |
| K24 | - (-) | 1024 (-) | - (-) | - (-) | -(-) | -(-) | -(-) | -(-) | -(-) | -(-) | 16(-) |
| K2 | - (-) | - (-) | - (-) | - (-) | -(-) | 1024(-) | -(-) | -(-) | 1024(-) | -(-) | 16(256) |
| ***Providencia stuartii*** |  |  |  |  |  |  |  |  |  |  |  |
| ATCC29914 | 256 (-) | 512 (-) | 1024 (-) | - (-) | -(-) | 512(-) | 1024(-) | -(-) | 1024(-) | -(-) | 8(128) |
| NAE 16 | 512 (-) | - (-) | - (-) | 1024 | -(-) | 256(1024) | 256(-) | -(-) | -(-) | -(-) | 8(256) |
| PS2636 | 512 (-) | 512 (-) | 1024 (-) | 512(1024) | -(-) | 256(-) | -(-) | -(-) | 512(-) | -(-) | 64(-) |
| PS299645 | 1024 (-) | - (-) | 1024 (-) | - (-) | -(-) | 1024(-) | -(-) | -(-) | 1024(1024) | -(-) | 32(-) |
| ***Pseudomonas aeruginosa*** |  |  |  |  |  |  |  |  |  |  |  |
| PA01 | - (-) | 1024 (-) | - (-) | 1024 | -(-) | 1024(-) | -(-) | ­-(-) | 1024(-) | 1024(-) | 16(256) |
| PA124 | - (-) | - (-) | - (-) | - (-) | -(-) | -(-) | -(-) | -(-) | 1024(-) | -(-) | 64(-) |

**Table S3.** Effects of different concentrations of plant extracts on the MIC (µg/ml) of antibiotics against *P. aeruginosa* PA124

| Plant extracts | | **Antibiotics** | | | | | | | | | |
| --- | --- | --- | --- | --- | --- | --- | --- | --- | --- | --- | --- |
|  |  | **CHL** | **AMP** | **CEF** |  | **KAN** | **NFX** | **ERY** | **CIP** | **TET** | **STR** |
|  | MIC antibiotic alone | 64 | - | - |  | 64 | 128 | 128 | 32 | 16 | 64 |
| ***Anthocleista schweinfurthii* (leaves)** | MIC/2 | 64 | - (na) | - (na) |  | **32(2)^S^** | 128(1)^I^ | **64(2)^S^** | 32(2)^S^ | 16(1)^I^ | 16(4)^S^ |
|  | MIC/5 | 64 | - (na) | - (na) |  | **32(2)^S^** | 128(1)^I^ | **64(2)^S^** | 32(2)^S^ | 16(1)^I^ | 16(4)^S^ |
|  | MIC/10 | 64 | - (na) | - (na) |  | **32(2)^S^** | 128(1)^I^ | **64(2)^S^** | 32(2)^S^ | 32(0.5)^I^ | **32(2)^S^** |
|  | MIC/20 | 64 | - (na) | - (na) |  | **32(2)^S^** | 128(1)^I^ | **64(2)^S^** | 32(2)^S^ | 32(0.5)^I^ | 32(2)^S^ |
| ***Anthocleista schweinfurthii (Fruits)*** | MIC/2 | 64 | - (na) | - (na) |  | **32(2)^S^** | 128(1)^I^ | 128(1)^I^ | **32(2)^S^** | 16(1)^I^ | **32(2)^S^** |
|  | MIC/5 | 64 | - (na) | - (na) |  | **32(2)^S^** | 128(1)^I^ | 128(1)^I^ | **32(2)^S^** | 16(1)^I^ | **32(2)^S^** |
|  | MIC/10 | 64 | - (na) | - (na) |  | 64(1)^I^ | 128(1)^I^ | 128(1)^I^ | **32(2)^S^** | 16(1)^I^ | **32(2)^S^** |
|  | MIC/20 | 64 | - (na) | - (na) |  | 64(1)^I^ | 128(1)^I^ | 128(1)^I^ | **32(2)^S^** | 16(1)^I^ | **32(2)^S^** |
| ***Anthocleista schweinfurthii (stem bark)*** | MIC/2 | 64 | - (na) | - (na) |  | 64(1)^I^ | 128(1)^I^ | 128(1)^I^ | **32(2)^S^** | 16(1)^I^ | **32(2)^S^** |
|  | MIC/5 | 64 | - (na) | - (na) |  | 64(1)^I^ | 128(1)^I^ | 128(1)^I^ | **32(2)^S^** | 16(1)^I^ | 64(1)^I^ |
|  | MIC/10 | 64 | - (na) | - (na) |  | 64(1)^I^ | 256(0,5)^I^ | 128(1)^I^ | **32(2)^S^** | 16(1)^I^ | 64(1)^I^ |
|  | MIC/20 | 64 | - (na) | - (na) |  | **64**(1)^I^ | 256(0,5)^I^ | 128(1)^I^ | **32(2)^S^** | 16(1)^I^ | 64(1)^I^ |
| ***Boehmeria platyphylla*** | MIC/2 | 64 | - (na) | - (na) |  | 64(1)^I^ | 128(1)^I^ | 128(1)^I^ | **32(2)^S^** | 16(1)^I^ | 64(1)^I^ |
|  | MIC/5 | 64 | - (na) | - (na) |  | 64(1)^I^ | 128(1)^I^ | 128(1)^I^ | **32(2)^S^** | 16(1)^I^ | 64(1)^I^ |
|  | MIC/10 | 64 | - (na) | - (na) |  | 64(1)^I^ | 128(1)^I^ | 128(0.5)^I^ | 32 | 16(1)^I^ | 64(1)^I^ |
|  | MIC/20 | 64 | - (na) | - (na) |  | 64(1)^I^ | 128(1)^I^ | 128(0.5)^I^ | 32 | 16(1)^I^ | 64(1)^I^ |

**Table S3 [continue].**

| **Plant extracts** | | **Antibiotics** | | | | | | | | | |
| --- | --- | --- | --- | --- | --- | --- | --- | --- | --- | --- | --- |
|  |  | **CHL** | **AMP** | **CEF** |  | **KAN** | **NFX** | **ERY** | **CIP** | **TET** | **STR** |
|  | MIC antibiotic alone | 64 | - | - |  | 64 | 128 | 128 | 32 | 16 | 64 |
| ***Caucalis melanantha*** | MIC/2 | 64(1)^I^ | - (na) | - (na) |  | 32(2)^S^ | 128(1)^I^ | 128(1)^I^ | 32(1)^I^ | 16(1)^I^ | **32(2)^S^** |
|  | MIC/5 | 64(1)^I^ | - (na) | - (na) |  | 64(1)^I^ | 128(1)^I^ | 128(1)^I^ | 32(1)^I^ | 16(1)^I^ | **32(2)^S^** |
|  | MIC/10 | 64(1)^I^ | - (na) | - (na) |  | 64(1)^I^ | 128(1)^I^ | 128(1)^I^ | 32(1)^I^ | 16(1)^I^ | **32(2)^S^** |
|  | MIC/20 | 64(1)^I^ | - (na) | - (na) |  | 64(1)^I^ | 128(1)^I^ | 128(1)^I^ | 32(1)^I^ | 16(1)^I^ | **32(2)^S^** |
| ***Erigeron floribundus*** | MIC/2 | 64(1)^I^ | - (na) | - (na) |  | **32(2)^S^** | 64(1)^I^ | 128(1)^I^ | **16(2)^S^** | **8(2)^S^** | **32(2)^S^** |
|  | MIC/5 | 64(1)^I^ | - (na) | - (na) |  | **32(2)^S^** | 64(1)^I^ | 128(1)^I^ | **16(2)^S^** | **8(2)^S^** | **32(2)^S^** |
|  | MIC/10 | 64(1)^I^ | - (na) | - (na) |  | **32(2)^S^** | 128(1)^I^ | 128(1)^I^ | **16(2)^S^** | **8(2)^S^** | **32(2)^S^** |
|  | MIC/20 | 64(1)^I^ | - (na) | - (na) |  | 64(1)^I^ | 128(1)^I^ | 128(1)^I^ | **16(2)^S^** | **8(2)^S^** | **32(2)^S^** |
| ***Laportea ovalifolia*** | MIC/2 | 64(1)^I^ | - (na) | - (na) |  | 64(1)^I^ | 128(1)^I^ | 128(1)^I^ | 32(1)^I^ | 16(1)^I^ | **32(2)^S^** |
|  | MIC/5 | 64(1)^I^ | - (na) | - (na) |  | 64(1)^I^ | 128(1)^I^ | 128(1)^I^ | 32(1)^I^ | 16(1)^I^ | **32(2)^S^** |
|  | MIC/10 | 64(1)^I^ | - (na) | - (na) |  | 64(1)^I^ | 128(1)^I^ | 128(1)^I^ | 32(1)^I^ | 16(1)^I^ | **32(2)^S^** |
|  | MIC/20 | 64(1)^I^ | - (na) | - (na) |  | 64(1)^I^ | 128(1)^I^ | 128(1)^I^ | 32(1)^I^ | 16(1)^I^ | **32(2)^S^** |

**Table S3 [continue and end].**

| **Plant extracts** | | **Antibiotics** | | | | | | | | | |
| --- | --- | --- | --- | --- | --- | --- | --- | --- | --- | --- | --- |
|  |  | **CHL** | **AMP** | **CEF** |  | **KAN** | **NFX** | **ERY** | **CIP** | **TET** | **STR** |
|  | MIC antibiotic alone | 64 | - | - |  | 64 | 128 | 128 | 32 | 16 | 64 |
| ***Nauclea latifolia* (leaves)** | MIC/2 | 64(1)^I^ | - (na) | - (na) |  | 64(1)^I^ | 128(1)^I^ | 128(1)^I^ | 32(1)^I^ | 1632(1)^I^ | 64(1)^I^ |
|  | MIC/5 | 64(1)^I^ | - (na) | - (na) |  | 64(1)^I^ | 128(1)^I^ | 128(1)^I^ | 32(1)^I^ | 1632(1)^I^ | 64(1)^I^ |
|  | MIC/10 | 64(1)^I^ | - (na) | - (na) |  | 64(1)^I^ | 128(1)^I^ | 128(1)^I^ | 32(1)^I^ | 1632(1)^I^ | 64(1)^I^ |
|  | MIC/20 | 64(1)^I^ | - (na) | - (na) |  | 64(1)^I^ | 128(1)^I^ | 128(1)^I^ | 32(1)^I^ | 1632(1)^I^ | 64(1)^I^ |
| ***Nauclea latifolia* (stem bark)** | MIC/2 | 64(1)^I^ | - (na) | - (na) |  | 64(1)^I^ | 128(1)^I^ | 128(1)^I^ | 32(1)^I^ | 1632(1)^I^ | 64(1)^I^ |
|  | MIC/5 | 64(1)^I^ | - (na) | - (na) |  | 64(1)^I^ | 128(1)^I^ | 128(1)^I^ | 32(1)^I^ | 1632(1)^I^ | 64(1)^I^ |
|  | MIC/10 | 64(1)^I^ | - (na) | - (na) |  | 64(1)^I^ | 128(1)^I^ | 128(1)^I^ | 32(1)^I^ | 1632(1)^I^ | 64(1)^I^ |
|  | MIC/20 | 64(1)^I^ | - (na) | - (na) |  | 64(1)^I^ | 128(1)^I^ | 128(1)^I^ | 32(1)^I^ | 1632(1)^I^ | 64(1)^I^ |
| ***Zehneria scobra*** | MIC/2 | 64(1)^I^ | - (na) | - (na) |  | 64(1)^I^ | 128(1)^I^ | 128(1)^I^ | 32(1)^I^ | 1632(1)^I^ | 64(1)^I^ |
|  | MIC/5 | 64(1)^I^ | - (na) | - (na) |  | 64(1)^I^ | 128(1)^I^ | 128(1)^I^ | 32(1)^I^ | 1632(1)^I^ | 64(1)^I^ |
|  | MIC/10 | 64(1)^I^ | - (na) | - (na) |  | 64(1)^I^ | 128(1)^I^ | 128(1)^I^ | 32(1)^I^ | 1632(1)^I^ | 64(1)^I^ |
|  | MIC/20 | 64(1)^I^ | - (na) | - (na) |  | 64(1)^I^ | 128(1)^I^ | 128(1)^I^ | 32(1)^I^ | 1632(1)^I^ | 64(1)^I^ |
| ***Nauclea latifolia* (fruits)** | MIC/2 | 64(1)^I^ | - (na) | - (na) |  | 64(1)^I^ | 128(1)^I^ | 128(1)^I^ | 32(1)^I^ | 16(1)^I^ | 64(1)^I^ |
|  | MIC/5 | 64(1)^I^ | - (na) | - (na) |  | 64(1)^I^ | 128(1)^I^ | 128(1)^I^ | 32(1)^I^ | 16(1)^I^ | 64(1)^I^ |
|  | MIC/10 | 64(1)^I^ | - (na) | - (na) |  | 64(1)^I^ | 128(1)^I^ | 128(1)^I^ | 32(1)^I^ | 16(1)^I^ | 64(1)^I^ |
|  | MIC/20 | 64(1)^I^ | - (na) | - (na) |  | 64(1)^I^ | 128(1)^I^ | 128(1)^I^ | 32(1)^I^ | 16(1)^I^ | 64(1)^I^ |

MIC: Minimal Inhibitory Concentration; -: >256 µg/ml; na : not applicable ; NT : not tested ; Antibotics (CHL: chloramphenicol, AMP : ampicillin, CEF: cefepime, KAN : kanamycin, NFX : norfloxacin, ERY : erythromycin, CIP : ciprofloxacin, TET : tetracycline, STR : streptomycin), (): fold decrease in MIC values of the antibiotics after association with plants extract; S: Synergy, I: Indifference.

**Table S4.** Effects of different concentrations of plant extracts on the MIC (µg/ml) of antibiotics against *E. aerogenes* CM64

| Plant extracts | | **Antibiotics** | | | | | | | | | |
| --- | --- | --- | --- | --- | --- | --- | --- | --- | --- | --- | --- |
|  |  | **CHL** | **AMP** | **CEF** |  | **KAN** | **NFX** | **ERY** | **CIP** | **TET** | **STR** |
| ***Anthocleista schweinfurthii* (leaves)** | MIC antibiotic alone | 512 | - | 256 |  | ≤2 | 2 | 256(1)^I^ | ≤0,5 | 32 | 16 |
| ***Anthocleista schweinfurthii* (leaves)** | MIC/2 | 512(1)^I^ | - (na) | 256(1)^I^ |  | ≤2(na) | 2(1)^I^ | 256(1)^I^ | ≤0,5(na) | 32(1)^I^ | **4(4)^S^** |
|  | MIC/5 | 512(1)^I^ | - (na) | 256(1)^I^ |  | ≤2(na) | 2(1)^I^ | 256(1)^I^ | ≤0,5(na) | 32(1)^I^ | 8**(2)^S^** |
|  | MIC/10 | 512(1)^I^ | - (na) | 256(1)^I^ |  | ≤2(na) | 2(1)^I^ | 256(1)^I^ | ≤0,5(na) | 32(1)^I^ | 16(1)^I^ |
|  | MIC/20 | 512(1)^I^ | - (na) | 256(1)^I^ |  | ≤2(na) | 2(1)^I^ | 256(1)^I^ | ≤0,5(na) | 32(1)^I^ | 16(1)^I^ |
| ***Anthocleista schweinfurthii* (fruits)** | MIC/2 | **256(2)^S^** | - (na) | NT |  | ≤2(na) | 2(1)^I^ | **128(2)^S^** | ≤0,5(na) | 16**(2)^S^** | 16(1)^I^ |
|  | MIC/5 | **256(2)^S^** | - (na) | NT |  | ≤2(na) | 2(1)^I^ | **128(2)^S^** | ≤0,5(na) | 16**(2)^S^** | 16(1)^I^ |
|  | MIC/10 | **256(2)^S^** | - (na) | NT |  | ≤2(na) | 2(1)^I^ | **128(2)^S^** | ≤0,5(na) | 16**(2)^S^** | 16(1)^I^ |
|  | MIC/20 | 512(1)^I^ | - (na) | NT |  | ≤2(na) | 2(1)^I^ | 256(1)^I^ | ≤0,5(na) | 16**(2)^S^** | 16(1)^I^ |
| ***Anthocleista schweinfurthii* (stem bark)** | MIC/2 | 512(1)^I^ | - (na) | NT |  | ≤2(na) | 2(1)^I^ | 256(1)^I^ | ≤0,5(na) | 32(1)^I^ | 16(1)^I^ |
|  | MIC/5 | 512(1)^I^ | - (na) | NT |  | ≤2(na) | 2(1)^I^ | 256(1)^I^ | ≤0,5(na) | 32(1)^I^ | 16(1)^I^ |
|  | MIC/10 | 512(1)^I^ | - (na) | NT |  | ≤2(na) | 2(1)^I^ | 256(1)^I^ | ≤0,5(na) | 32(1)^I^ | 16(1)^I^ |
|  | MIC/20 | 512(1)^I^ | - (na) | NT |  | ≤2(na) | 2(1)^I^ | 256(1)^I^ | ≤0,5(na) | 32(1)^I^ | 16(1)^I^ |
| ***Boehmeria platyphylla*** | MIC/2 | 512(1)^I^ | - (na) | NT |  | ≤2(na) | 2(1)^I^ | 256(1)^I^ | ≤0,5(na) | 16**(2)^S^** | 4**(4)^S^** |
|  | MIC/5 | 512(1)^I^ | - (na) | NT |  | ≤2(na) | 2(1)^I^ | 256(1)^I^ | ≤0,5(na) | 16**(2)^S^** | 4**(4)^S^** |
|  | MIC/10 | 512(1)^I^ | - (na) | NT |  | ≤2(na) | 2(1)^I^ | 256(1)^I^ | ≤0,5(na) | 16**(2)^S^** | 8**(2)^S^** |
|  | MIC/20 | 512(1)^I^ | - (na) | NT |  | ≤2(na) | 2(1)^I^ | 256(1)^I^ | ≤0,5(na) | 16**(2)^S^** | 16(1)^I^ |

**Table S4 [continue].**

| **Plant extracts** | | **Antibiotics** | | | | | | | | | |
| --- | --- | --- | --- | --- | --- | --- | --- | --- | --- | --- | --- |
|  |  | **CHL** | **AMP** | **CEF** |  | **KAN** | **NFX** | **ERY** | **CIP** | **TET** | **STR** |
|  | MIC antibiotic alone | 512 | - | 256 |  | ≤2 | 2 | 256 | ≤0,5 | 32 | 16 |
| ***Caucalis melanantha*** | MIC/2 | 512(1)^I^ | - (na) | - (na) |  | ≤2(na) | 2(1)^I^ | 256(1)^I^ | ≤0,5(na) | 32(1)^I^ | 16(1)^I^ |
|  | MIC/5 | 512(1)^I^ | - (na) | - (na) |  | ≤2(na) | 2(1)^I^ | 256(1)^I^ | ≤0,5(na) | 32(1)^I^ | 16(1)^I^ |
|  | MIC/10 | 512(1)^I^ | - (na) | - (na) |  | ≤2(na) | 2(1)^I^ | 256(1)^I^ | ≤0,5(na) | 32(1)^I^ | 16(1)^I^ |
|  | MIC/20 | 512(1)^I^ | - (na) | - (na) |  | ≤2(na) | 2(1)^I^ | 256(1)^I^ | ≤0,5(na) | 32(1)^I^ | 16(1)^I^ |
| ***Erigeron floribundus*** | MIC/2 | 512(1)^I^ | - (na) | NT |  | ≤2(na) | **1(2)^S^** | **128(2)^S^** | ≤0,5(na) | 16**(2)^S^** | 16(1)^I^ |
|  | MIC/5 | 512(1)^I^ | - (na) | NT |  | ≤2(na) | **1(2)^S^** | 256(1)^I^ | ≤0,5(na) | 16**(2)^S^** | 16(1)^I^ |
|  | MIC/10 | 512(1)^I^ | - (na) | NT |  | ≤2(na) | **1(2)^S^** | 256(1)^I^ | ≤0,5(na) | 32(1)^I^ | 16(1)^I^ |
|  | MIC/20 | 512(1)^I^ | - (na) | NT |  | ≤2(na) | 2(1)^I^ | 256(1)^I^ | ≤0,5(na) | 32(1)^I^ | 16(1)^I^ |
| ***Laportea ovalifolia*** | MIC/2 | 512(1)^I^ | - (na) | NT |  | ≤2(na) | 2(1)^I^ | 256(1)^I^ | ≤0,5(na) | 32(1)^I^ | 16(1)^I^ |
|  | MIC/5 | 512(1)^I^ | - (na) | NT |  | ≤2(na) | 2(1)^I^ | 256(1)^I^ | ≤0,5(na) | 32(1)^I^ | 16(1)^I^ |
|  | MIC/10 | 512(1)^I^ | - (na) | NT |  | ≤2(na) | 2(1)^I^ | 256(1)^I^ | ≤0,5(na) | 32(1)^I^ | 16(1)^I^ |
|  | MIC/20 | 512(1)^I^ | - (na) | NT |  | ≤2(na) | 2(1)^I^ | 256(1)^I^ | ≤0,5(na) | 32(1)^I^ | 16(1)^I^ |

**Table S4 [continue and end].**

| **Plant extracts** | | **Antibiotics** | | | | | | | | | |
| --- | --- | --- | --- | --- | --- | --- | --- | --- | --- | --- | --- |
|  |  | **CHL** | **AMP** | **CEF** |  | **KAN** | **NFX** | **ERY** | **CIP** | **TET** | **STR** |
|  | MIC antibiotic alone | 512 | - | 256 |  | ≤2 | 2 | 256 | ≤0,5 | 32 | 16 |
| ***Nauclea latifolia* (leaves)** | MIC/2 | 512(1)^I^ | - (na) | NT |  | ≤2(na) | 2(1)^I^ | **128(2)^S^** | ≤0,5(na) | **16(2)^S^** | **8(2)^S^** |
|  | MIC/5 | 512(1)^I^ | - (na) | NT |  | ≤2(na) | 2(1)^I^ | 256(1)^I^ | ≤0,5(na) | **16(2)^S^** | 16(1)^I^ |
|  | MIC/10 | 512(1)^I^ | - (na) | NT |  | ≤2(na) | 2(1)^I^ | 256(1)^I^ | ≤0,5(na) | **16(2)^S^** | 16(1)^I^ |
|  | MIC/20 | 512(1)^I^ | - (na) | NT |  | ≤2(na) | 2(1)^I^ | 256(1)^I^ | ≤0,5(na) | **16(2)^S^** | 16(1)^I^ |
| ***Nauclea latifolia* (fruits)** | MIC/2 | 512(1)^I^ | - (na) | 256(1)^I^ |  | ≤2(na) | 2(1)^I^ | 256(1)^I^ | ≤0,5(na) | 32(1)^I^ | 4**(4)^S^** |
|  | MIC/5 | 512(1)^I^ | - (na) | 256(1)^I^ |  | ≤2(na) | 2(1)^I^ | 256(1)^I^ | ≤0,5(na) | 32(1)^I^ | 4**(4)^S^** |
|  | MIC/10 | 512(1)^I^ | - (na) | 256(1)^I^ |  | ≤2(na) | 2(1)^I^ | 256(1)^I^ | ≤0,5(na) | 32(1)^I^ | 16(1)^I^ |
|  | MIC/20 | 512(1)^I^ | - (na) | 256(1)^I^ |  | ≤2(na) | 2(1)^I^ | 256(1)^I^ | ≤0,5(na) | 32(1)^I^ | 16(1)^I^ |
| ***Nauclea latifolia (stem bark)*** | MIC/2 | 512(1)^I^ | - (na) | 256(1)^I^ |  | ≤2(na) | 2(1)^I^ | 256(1)^I^ | ≤0,5(na) | **16(2)^S^** | 2**(4)^S^** |
|  | MIC/5 | 512(1)^I^ | - (na) | 256(1)^I^ |  | ≤2(na) | 2(1)^I^ | 256(1)^I^ | ≤0,5(na) | **16(2)^S^** | **8(2)^S^** |
|  | MIC/10 | 512(1)^I^ | - (na) | 256(1)^I^ |  | ≤2(na) | 2(1)^I^ | 256(1)^I^ | ≤0,5(na) | **16(2)^S^** | 16(1)^I^ |
|  | MIC/20 | 512(1)^I^ | - (na) | 256(1)^I^ |  | ≤2(na) | 2(1)^I^ | 256(1)^I^ | ≤0,5(na) | 32(1)^I^ | 16(1)^I^ |
| ***Zehneria scobra*)** | MIC/2 | 512(1)^I^ | - (na) | 256(1)^I^ |  | ≤2(na) | 2(1)^I^ | **128(2)^S^** | ≤0,5(na) | 32(1)^I^ | 4**(4)^S^** |
|  | MIC/5 | 512(1)^I^ | - (na) | 256(1)^I^ |  | ≤2(na) | 2(1)^I^ | 256(1)^I^ | ≤0,5(na) | 32(1)^I^ | 4**(4)^S^** |
|  | MIC/10 | 512(1)^I^ | - (na) | NT |  | ≤2(na) | 2(1)^I^ | 256(1)^I^ | ≤0,5(na) | 32(1)^I^ | **8(2)^S^** |
|  | MIC/20 | 512(1)^I^ | - (na) | NT |  | ≤2(na) | 2(1)^I^ | 256(1)^I^ | ≤0,5(na) | 32(1)^I^ | **8(2)^S^** |

MIC: Minimal Inhibitory Concentration; -: >256 µg/ml; na : not applicable ; NT : not tested ; Antibotics [CHL: chloramphenicol, AMP : ampicillin, CEF: cefepime, KAN : kanamycin, NFX : norfloxacin, ERY : erythromycin, CIP : ciprofloxacin, TET : tetracycline, STR : streptomycin, (): fold decrease in MIC values of the antibiotics after association with plants extract; S: Synergy, I: Indifference.

**References**

1. Viveiros M, Jesus A, Brito M, Leandro C, Martins M, Ordway D, Molnar M, Molnar J, Amaral L: **Inducement and reversal of tetracycline resistance in *Escherichia coli* K-12 and expression of proton gradient-dependent multidrug efflux pump genes**. *Antimicrob Agents Chemother* 2005, **49**(8):3578-3582.

2. Okusu H, Ma D, Nikaido H: **AcrAB efflux pump plays a major role in the antibiotic resistance phenotype of *Escherichia coli* multiple-antibiotic- resistance Mar mutants.** *J Bacteriol* 1996, **178**(1):306-308.

3. Elkins CA, Mullis LB: **Substrate competition studies using whole-cell with the major tripartite multidrug efflux pumps of *Escherichia coli***. *Antimicrob Agents Chemother* 2007, **1**(3):923-929.

4. Baglioni P, Bini L, Liberatori S, Pallini V, Marri L: **Proteome analysis of *Escherichia coli* W3110 expressing an heterologous sigma factor**. *Proteomics* 2003, **3**(6):1060-1065.

5. Sar C, Mwenya B, Santoso B, Takaura K, Morikawa R, Isogai N, Asakura Y, Toride Y, Takahashi JA: **Effect of *Escherichia coli* wild type or its derivative with high nitrite reductase activity on in vitro ruminal methanogenesis and nitrate/nitrite reduction**. *J Anim Sci* 2005, **83**:644-652.

6. Ghisalberti D, Masi M, Pagès J-M, Chevalier J: **Chloramphenicol and expression of multidrug efflux pump in *Enterobacter* *aerogenes***. *Biochem Biophys Res Commun* 2005, **328**(4):1113-1118.

7. Malléa M, Mahamoud A, Chevalier J, Alibert-Franco S, Brouant P, Barbe J, Pagès JM: **Alkylaminoquinolines inhibit the bacterial antibiotic efflux pump in multidrug-resistant clinical isolates**. *Biochem J* 2003, **376**:801-805.

8. Pradel E, Pagès J-M: **The AcrAB-TolC efflux pump contributes to multidrug resistance in the nosocomial pathogen *Enterobacter aerogenes*** *Antimicrob Agents Chemother* 2002, **46**(2640-2643).

9. Chevalier J, Pagès J-M, Eyraud A, Malléa M: **Membrane Permeability Modifications Are Involved in Antibiotic Resistance in *Klebsiella pneumoniae***. *Biochem Biophys Res Commun* 2000, **274**(2):496-499.

10. Tran Q-T, Mahendran KR, Hajjar E, Ceccarelli M, Davin-Regli A, Winterhalter M, Weingart H, Pagès J-M: **Implication of Porins in β-Lactam Resistance of *Providencia stuartii***. *J Biol Chem* 2010, **285**(42):32273–32281.

11. Lorenzi V, Muselli A, Bernardini AF, Berti L, Pagès JM, Amaral L, Bolla JM: **Geraniol restores antibiotic activities against multidrug-resistant isolate from Gram-negative species.** .*Antimicrob Agents Chemother* 2009, **53**: 2209-2211.
